# Type II acquired cutis laxa associated with recurrent urticarial vasculitis: brief report

**DOI:** 10.1186/s13223-019-0401-y

**Published:** 2020-01-03

**Authors:** Amelia Nabatanzi, Siqi Da, Musa Male, Siyuan Chen, Changzheng Huang

**Affiliations:** 1 Department of Dermatology, Union Hospital, Tongji Medical College, Huazhong University of Science and Technology, Wuhan, 430022 China; 2 Department of Urology, Tongji Hospital, Tongji Medical College, Huazhong University of Science and Technology, Wuhan, China

**Keywords:** Acquired cutis laxa, Urticarial vasculitis, Elastolysis activity, Dermal elastin

## Abstract

**Background:**

Cutis laxa is a connective tissue disease characterized by loose, wrinkled, and redundant skin. It is either inherited or acquired. In most cases, acquired cutis laxa is associated with neoplasms, drugs, and autoimmune diseases. We present a rare case of acquired cutis laxa following a recurrent urticaria-like eruption in the absence of an autoimmune disease, neoplasm, drugs and or syndrome.

**Case presentation:**

We report a case of a 45-year-old Chinese lady with a 1-year history of widespread pruritic urticarial eruption and a 6-month history of progressive skin wrinkling. On examination, the patient appeared older than her actual age, with apparent wrinkling on the mid-torso with generalized smooth, erythematous macules and wheals. A family history of similar conditions was absent. Biopsy revealed hypersensitivity and atrophy. Following the Food and Drug Administration (FDA) guidelines, we administered antihistamines, which relieved the itching, but her hyperpigmentation and cutis laxa never improved.

**Conclusion:**

Our case shows that the decrease of elastic fibers may be associated with the infiltration of inflammatory cells in the dermis. This supports the hypothesis that chemical mediators may play a major role in the destruction of elastic fibers, thus causing cutis laxa. In addition, we advise practitioners to take a complete clinical and family history to determine if the condition is inherited or acquired.

## Background

Acquired cutis laxa is a phenotype of cutis laxa affecting dermal elastin [1]. It has two forms; type I and type II. Type I can either be localized or generalized involving visceral organs, such as respiratory, cardiovascular, gastrointestinal, and genitourinary systems [2, 3]; elasticity of the skin in type I can be observed both in inflammatory and non-inflammatory areas. Type II is localized post-inflammatory elastolysis, where skin elasticity occurs over the inflammatory area without involving visceral organs [4, 5]. It is seen mostly in adults; onset is abrupt mainly with a cephalocaudal predisposition [3]. We present a case of a 45-year-old Chinese lady with progressing skin wrinkling after the urticaria-like eruption. To our knowledge, this is the first case documenting acquired cutis laxa following urticarial vasculitis in the absence of an autoimmune disease, neoplasm, drugs, and syndrome.

## Case presentation

Our patient, a 45-year-old Chinese lady, was healthy up until February 2017, when she presented to a clinic in her hometown with generalized, pruritic, mildly painful, erythematous macules and wheals all over her body. They noted lesions persisted for 2–3 days and left hyperpigmented marks on disappearing. They diagnosed her with chronic urticaria, which was controlled with oral antihistamines. Around October 2017, her skin had become atrophic, loose, and sagging, specifically around her mid-torso region. The local clinic continued her on antihistamines for the urticarial wheals, and they advised her that once urticarial wheals improved, the skin would go back to normal. With no change in symptoms, the patient was then referred to our hospital.

**Fig. 1:**
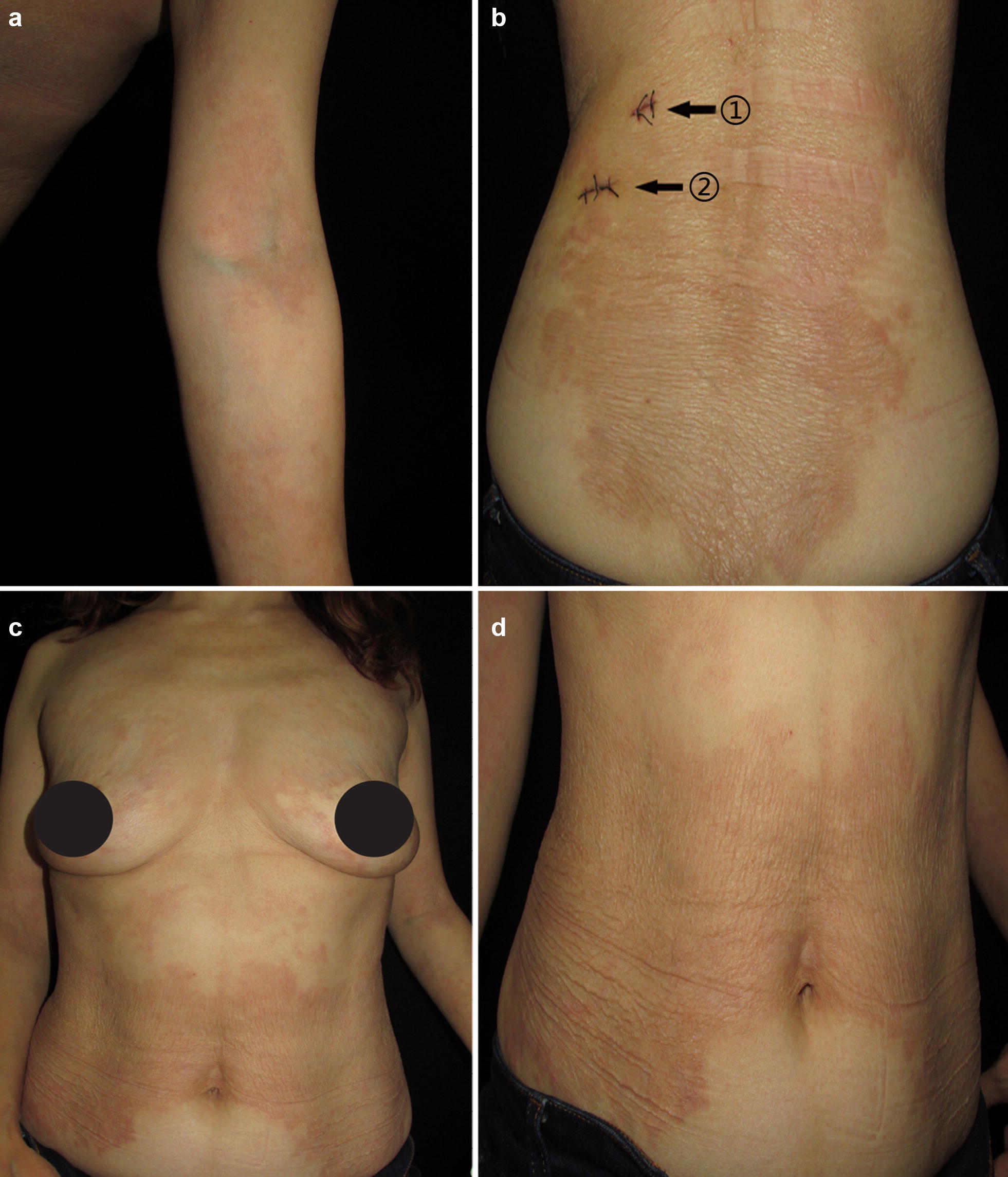
Showing clinical picture. **a** Erythematous wheals on the elbow, extending towards the upper arm. **b**–**d** Skin atrophy, wrinkles, and hyperpigmentation on the lateral and anterior side of the torso, respectively. ① and ②, sites for biopsy

The patient came to us in March 2018, with a chief complaint of a 1-year history of widespread pruritic urticarial eruption and a 6-month history of progressive skin wrinkling, which gave her an aged appearance. She reported no family history of a similar condition. On examination, the 45-year-old lady appeared older than her actual age, with loose, saggy, and wrinkled skin on the anterior and posterior trunk. She had multiple 2–5 cm of smooth, erythematous wheals on the upper extremities, and extensive skin atrophy and hyperpigmentation on the trunk (Fig. 1). She had unilateral right supraclavicular (14.4 * 5.6 mm), bilateral cervical (R = 19.9 * 5.4 mm, L = 13.4 * 5.9 mm), auxiliary (R = 14.4 * 4.6 mm, L = 16.2 * 6.7 mm) and inguinal (R = 27.9 * 4.1 mm, L = 17.0 * 4.1 mm) swelling of lymph nodes. No fever, joint and bone pain.

**Fig. 2:**
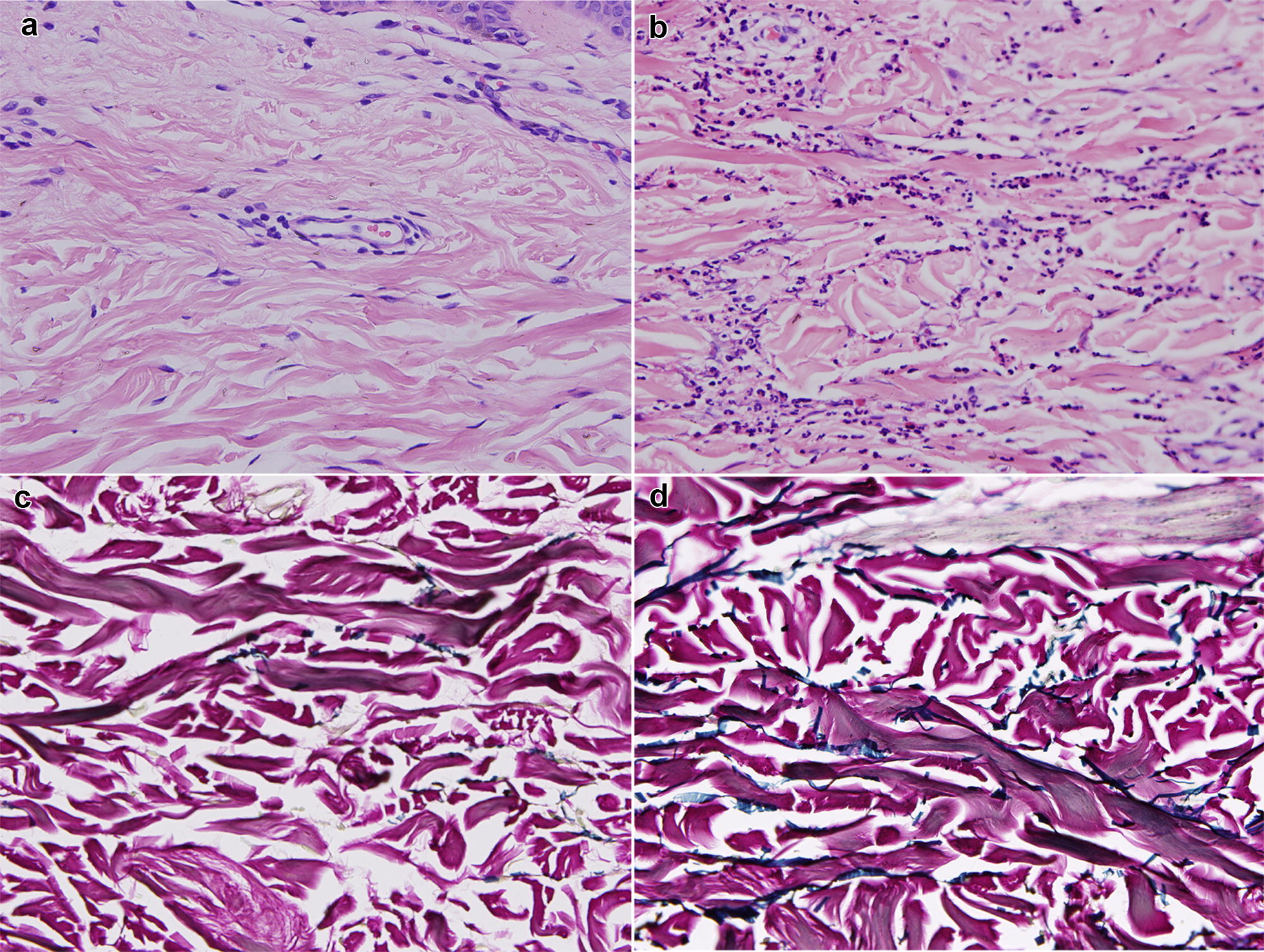
Showing histology findings. **a** (From biopsy site **①**) and **b** (from biopsy site **②**), multiple neutrophils, with few eosinophils and no edema with deposition of elastin and collagen bundle fragmentation in deep dermis (HE × 200). **c** (From biopsy site **①**) and **d** (from biopsy site **②**) dispersed elastin showing an amorphous substance and fragmentation of elastic fibers positive with Verhoeff-van Gieson stain (× 400)

Incision biopsy for hematoxylin–eosin (HE) and Verhoeff-van Gieson stain was done from the right-side flank and showed neutrophilic infiltration of the interstitial dermis and extravasated erythrocytes. Biopsy also revealed decreased elasticity of dermal tissue with clustered fibers or fragmented with infiltrated inflammatory changes in collagen. There was a breakdown of erythrocytes, peripheral nerve infiltration, deposition of elastin, and separation of collagen bundles confirmed the diagnosis (Fig. 2).

Additional examination included; blood test: Neutrophils = 38.5% (normal range is 40–75%), lymphocytes = 54.9% (normal range is 20–50%). White blood cell 4.06 G/L (normal range is 3.5–9.5 g/L), alpha antitrypsin (109 mg/mL), urine test, fecal test, ESR, liver, and kidney function tests, tb-ab, T-SPOT, ANA test, ANCA, and serum complement test (s) were normal. IgG 7.36 g/L (normal range 7.3–15.6 g/L), IgA I.35 (normal range 0.82–4.53 g/L), IgE 191.6 IU/mL (normal range 1–190 IU/mL), IgM 8.91 g/L (normal range 0.46–3.04 g/L), CRP 18.9 mg/L (normal range is < 8.00 mg/L) and KTG test (167.2).)

## Discussion and conclusion

Acquired cutis laxa may be associated with autoimmune diseases such as systemic lupus erythematosus, inflammatory processes including chronic urticaria, and urticarial vasculitis, as well as reduced alpha antitrypsin [5, 6]. The proposed mechanism by which these factors are associated with acquired cutis laxa revolves around the loss of elastic fibers [1, 7]. An immune-mediated mechanism has also been postulated to cause cutis laxa [4, 8], in which case the activated neutrophils produce elastase, which breaks down collagen fibers and induces elastolysis [7, 9]. Our patients had elevated levels of CRP, IgE, and IgM. Elevated IgE is consistent with an allergic reaction, while elevated CRP and IgM are consistent with an acute infection that induced lymphadenopathy. Additionally, histopathology showed neutrophilic infiltration between collagen fibers and leukocytoclasia around blood vessels, which are the characteristic features of acquired cutis laxa [1]. According to published literature, autoimmune diseases deposit immunoglobulins in the dermis, which causes the destruction of elastic tissue resulting in acquired cutis laxa [3, 10]; however, in this case, the acquired cutis laxa followed an inflammatory condition.

A few authors reported acquired cutis laxa after urticarial vasculitis; however, it was in the setting of an autoimmune disease such as systemic lupus erythematosus, IgA myeloma [3, 11]; but our patient presented with loose, saggy, and wrinkled skin on the anterior and posterior trunk without systemic symptoms. Although these are symptoms of cutis laxa, they can overlap with other diseases such as mid-dermal elastolysis, which presents with erythema reticulated plaques with patches of fine wrinkling [12]. Granulomatous slack skin may also present in the same way as acquired cutis laxa, but it is characterized by painless purple plaques and redundant sagging skin [13].

The relationship between acquired cutis laxa and urticaria-like eruption is still not clear. Moreover, Fornieri C et al. stated that the increase in protein elastin turn-over and reduction of serum alpha antitrypsin by fibroblasts increases elastolytic activity [1]. However, our case differs from Fornieri et al. findings since the acquired cutis laxa occurred after urticaria-like skin lesions without an underlying systemic condition. Therefore, we propose that future research should focus on chemical mediators involved in the breakdown of elastic fibers, which causes elastolysis. Finally, it’s crucial to get a complete history to determine if the condition is systemic or acquired.

## Data Availability

All data and materials can be obtained by email to the corresponding author.

## Abbreviations

ESR: erythrocyte sedimentation rate
T-SPOT: tuberculosis-spot
ANA: antinuclear antibody
ANCA: antineutrophil cytoplasmic antibodies
SLE: systemic lupus erythematosus
IgG: immunoglobulin G
IgM: immunoglobulin M
IgA: immunoglobulin A
CRP: C-reactive protein
KTG: kinetic turbidimetric (1,3) beta-d-glucan detection
HE: hematoxylin–eosin
